# Comparison of Cattle Housing Systems Based on the Criterion of Damage to Barn Equipment and Construction Errors

**DOI:** 10.3390/ani12192530

**Published:** 2022-09-22

**Authors:** Marek Gaworski, Michał Boćkowski

**Affiliations:** 1 Department of Production Engineering, Institute of Mechanical Engineering, Warsaw University of Life Sciences, 02-787 Warsaw, Poland; 2 Metal Structures Company, 07-300 Ostrów Mazowiecka, Poland

**Keywords:** barn, construction error, dairy cattle, damage, freestall housing system, tie-stall housing system

## Abstract

**Simple Summary:**

As a result of many years of use, dairy cattle barns are subject to gradual wear and degradation. Damage to technical equipment can be identified in many areas in the barn. These areas are used by dairy cattle, so it is important to recognize the problem of damage and the associated health risks for animals. The problem of damage to internal equipment (e.g., damage to the floor, partitions between lying stalls, feed ladders, drinking bowls) applies to both tie-stall and freestall barns, which are the most common in dairy farms. Such premises became an inspiration to compare barns with a tie-stall system, a freestall system and their individual areas (lying, feeding, milking and social) in terms of the amount of damage but also construction errors. Most damage per one barn was found in the feeding area of objects with a tie-stall housing system. More cow health problems (e.g., laminitis, hoof problems) were identified in the barns with the freestall housing system. Equipment failures and construction errors may disrupt efficient and animal-safe dairy production in the barn. The results of the research study may be an incentive for farmers to check the barns in terms of their technical wear.

**Abstract:**

Dairy cattle housing systems are the subject of numerous studies, in which a strong emphasis is placed on the comparison of animal welfare, animal behavior, production indicators and labor inputs. Dairy cattle housing systems are linked to specific livestock buildings, which is a prerequisite for undertaking studies comparing barns and their technical equipment. The aim of the study was to compare barns with two types of housing systems, i.e., tie-stall and freestall, including the identification of technical wear in various areas used by animals. This objective was linked to the assessment of animal health problems in livestock facilities. The research covered 38 dairy farms, 19 of which kept cows in the tie-stall system and 19 in the freestall system. The barns in these farms were examined for technical damage and construction errors, assessed in four areas: lying, feeding, milking and social. The research results confirmed significant differences in the degree of damage to technical equipment in individual areas of barns and between barns with tie-stall and freestall housing systems. The conclusions indicate the need to link the degradation of barns and their technical equipment, as well as design errors with the evaluation of dairy cattle welfare in future studies.

## 1. Introduction

Dairy cattle barns and production technologies implemented in the barn are the subject of many evaluations by farmers, advisory services and research teams. The assessments concern the design of the barn, its structure and functionality, technical equipment, the degree of mechanization and automation of work, lighting, thermal and microclimatic conditions, which determine sustainable production in terms of environmental, economic and social conditions [1]. Cattle in the barn, their comfort and well-being in various housing systems are also assessed by farmers and advisory services, for example, in order to obtain subsidies for the improvement of living conditions. The working conditions of the people who manage the herd of animals and perform tasks in dairy production technology are also important issues, and these conditions should be considered in the assessment of the barn. In many farms, cows spend the whole year in the barn, which is why this facility requires special attention in the assessment to ensure a high level of comfort and safety for the animals.

A barn is a building where dairy cows should be able to live in a sustainable production environment [2], which is necessary for animal health and welfare [3]. According to Hughes [4], animal welfare generally refers to a state of full mental and physical health in which the animal is in close harmony with the environment. Hurnik and Lehman [5] indicate that animal welfare is a state of physical and mental harmony between the body and the environment. Both the environmental conditions and the technical equipment that make up the housing environment are key components of animal production. Therefore, their inclusion in research is a valuable contribution to improving animal welfare.

Knowledge of available technical equipment and requirements for environmental conditions in the barn are important factors at the barn design stage for each of the dairy cattle housing systems. The basic criterion for designing a building for dairy cattle is to create an optimally healthy and friendly environment for cattle [6]. Barn equipment must ensure the well-being of cows, which is a key objective in the design of dairy barns [7].

The role of barn technical equipment and its importance in dairy production technology on farms have been developed in many experiments focused on particular areas of the barn, where dairy cattle activity is analyzed to find conditions favorable to animal comfort and welfare. The results of some studies in the lying area emphasize the importance of neck-rail location, characteristic for barns with the freestall housing system. The location of the neck-rail can change the way cows stand in the stall [8] and have an impact on the preferences [9], use and cleanliness of the stall [10]. Much technical data relates to the state of the surface for lying dairy cattle in a barn with a tie-stall and freestall housing system. The thickness of mats/mattresses covering lying stalls and their individual features (surface shape, softness, water absorption, anti-slip properties) determine the comfort of animals in the barn [11]; the rubber surface may affect the behavior of dairy cows, including sick cows [12]. Drissler et al. [13] stated that lowering the level of bedding material (sand) in the lying area reduces the time spent lying by cows. Research conducted by Fregonesi et al. [14] showed the impact of bedding material quality, i.e., sand moisture on cow preferences and lying time. In addition to sand, the research covers other, alternative bedding materials, and the recognition of the properties of these materials allows for their proper selection in terms of the welfare requirements of dairy cattle [15]. When assessing the lying area, studies with cows also take into account the features of other technical equipment in barns, including stall partitions (typical for a tie-stall and freestall system) [16] and brisket board (typical for a freestall system) [17].

Additionally, some studies in other areas of the barn show the impact of technical solutions on animal preferences, health problems, animal comfort/discomfort, and some production indicators. The role of the feed barrier design (used in barns with tie-stall and freestall system) in the behavior of dairy cows has been studied by Endres et al. [18] and developed by Huzzey et al. [19], who examined the feed barrier design in relation to the stocking density to analyze the feeding and social behavior of dairy cattle. Appropriate design of the feeding area for cattle translates into the available space for animals in the place where the feed is taken, and as a result, their behavior and possible aggression, which affects the welfare [20].

Good water supply is extremely important for high-yielding dairy cows, which is why some research has been developed to investigate how cattle respond to differences in water supply, including the use of specialized technical equipment both in the barn with tie-stall and freestall system. By designing better drinking troughs in the barn, Machado Filho et al. [21] found that dairy cows prefer to drink and drink more from larger troughs, while Teixeira et al. [22] indicated that the surface and height, but not the depth of the drinking trough, affect dairy cows’ preferences. The importance of properly designed drinkers in the barn is especially noticeable in hot weather, when competition between cows regarding access to water is increasing [23].

Specialized technical equipment is a key element in the planning of the milking center on dairy farms with the freestall housing system [24]. On dairy farms with different types of milking systems, research is carried out to compare technical performance [25] and compare dairy herd assessment indicators [26]. The impact of automatic milking systems on the management, behavior, health and welfare of dairy cows [27], as well as the design of automated milking cows to maximize work efficiency [28], shows the relationship between modern milking equipment and some factors taken into account in the assessment of dairy production in the barn. The potential of automatic milking systems allows farmers to increase the efficiency of the milk production process and sustainable animal welfare [29]. Proper design of the milking area is important for both cows and the farmer [30], taking into account the producer’s satisfaction with the applied and evaluated milking system [31], both in the barn with a tie-stall system and one with a freestall system.

The quality of the flooring is of great importance in the barn. The quality of the floor is emphasized in pens with a freestall system, in barns with a tie-stall system, but also in a maternity area. In research by Campler et al. [32] on the basis of observed lying times and lying bouts, it was noticed that cows avoid a rubber floor in the maternity pen. The floor as a key element of the barn design is an area of many studies that assess the interactions between some floor features and cattle, both in the barn with tie-stall and freestall system. One of the research objectives is to compare different types of floors and their impact on the behavior of dairy cows [33]. Comparing concrete floors with a more compressible surface, such as rubber, it was observed that cows spent more time standing [34] and showed preferences for rubber [35]. Cattle choose to walk on floors that are not only soft [36] but also provide good traction and friction [37]. When assessing the floor of the barn, the research took into account such features as surface slipperiness and floor compressibility [38]. There are questions about the barn floor, such as [39], do cows prefer a compartment with a grooved or slotted floor? Therefore, studies are undertaken to assess the function of common solid solutions to improve the traction of cattle flooring [40]. Barn floor quality can be an indicator of animal health. Poor flooring increases the risk of impaired locomotion [41] and injuries [42], so some links can be confirmed between the floor and the assessment of dairy cattle health problems.

Technical facilities also play a key, responsible role in the barn when it comes to maintaining appropriate microclimatic conditions in each barn with tie-stall and freestall system. Comparing different ventilation systems in a dairy building provides information on energy consumption, running costs and some suggestions, e.g., fan selection is a critical design element [43]. Effective technical support of microclimatic conditions in the barn, especially important in terms of heat stress in cows [44,45], depends on some equipment properties and technical condition [46].

The technical equipment responsible for access to light in the barn with tie-stall and freestall system, in conjunction with the role of photoperiod in dairy production [47], translates into cows’ behavior in various places in the barn and production-related indicators [48].

Various criteria are used to evaluate the technical equipment of a barn. The general evaluation criteria include the reliable operation and safe operation of technical equipment, while the detailed criteria take into account the fulfillment of the requirements in terms of technical parameters, standards, efficiency and quality of the construction materials used. Taking into account the direct contact of animals with the technical equipment of the barn, it seems important to assess the condition of the barn equipment and whether it is not harmful to the animals.

During many years of operation, livestock buildings and their equipment are subject to gradual wear and degradation. Damaged equipment, deteriorated surface (concrete) of the feed alleys, lying area and social area, as well as certain design errors may pose risks in contact with animals, hence the need to monitor and—if necessary—repair and supplement the technical equipment. Knowledge of technical problems in buildings with tie-stall and freestall housing system can be an additional argument when comparing these systems.

The aim of the study was to compare barns with two types of housing systems, i.e., tie-stall and freestall, including the identification of technical wear in various areas used by animals. This objective was linked to the assessment of animal health problems in livestock facilities.

The intention of comparing barns with tie-stall and freestall systems is to show differences in terms of identified technical problems and design errors. These differences concern, for example, the detailed type of damage, the number of possible types of damage to technical equipment and types of construction errors in tie-stall and freestall barns. As a result of testing the barns with the tie-stall system and the freestall system, it is possible to indicate which errors can be avoided in the design of barns and in which places of both types of barn technical problems should be considered. Showing technical problems in tie-stall and freestall barns allows us to raise farmers’ awareness when choosing a cattle housing system on the farm.

## 2. Materials and Methods

Two regions of Poland, i.e., Mazovia and Podlasie, were selected for the evaluation of the technical condition of barn equipment. These regions, located in central and eastern Poland, have a high dairy production potential, especially access to a large area of grassland compared to the rest of the country. These regions are also characterized by a large number of dairy farms, differing in the number of cows in the herd and the dairy housing systems used. According to the data of the Statistics Poland [49] for June 2021, 18.2% of all cows in Poland were kept in the Mazovia Voivodeship, and 16.8% in the Podlasie Voivodeship; the territory of Poland is divided into 16 voivodships, and the Mazovia and Podlasie voivodships cover a total of 17.8% of the country’s area. In 2020, the voivodeships selected for research were also distinguished by the highest milk production in Poland per 1 ha of arable land, 1574 L/ha and 2680 L/ha in the Mazovia and Podlasie voivodeships, respectively; the average for Poland was 981 l/ha. The annual milk yield of cows in both regions was slightly differentiated (5990 L/year in Mazovia and 6228 L/year in Podlasie) and higher than the average for Poland (5946 L/year). The potential of the dairy regions selected for the study is confirmed by the fact that together, 41.3% of the total amount of milk produced in Poland in 2020 came from the Mazovia and Podlasie voivodeships [50].

On the basis of the available list of over 150 dairy farms known from the cooperation so far, we randomly selected 40 farms for the study. The owners of the farms were contacted by phone to obtain their consent to conduct the research. In addition, in discussions with the owners, an outline of the research plan and its purpose were presented. If the farm owner agreed to the research, details of the visit were arranged. In a situation where there was no consent from the owner to visit, another farm was selected at random. At the stage of selecting farms for the research, the principle was followed to include the same number of objects in each of the considered cow housing systems. Finally, a group of 38 family dairy farms were selected for the evaluation of the barns, including 19 with tie-stall housing system and 19 with freestall housing system in barns. In practice, the approach to evaluate the sample needed for accurate estimation of outcome-based measurements [51] is known. In our research, when selecting the sample size (number of farms), we followed the principle of its representativeness in relation to the number of dairy farms in a given region. Dairy farms differed in the size of the herd (Table 1) and in some details of the barns and their technical facilities. Each farm had one barn which was carefully assessed. For the purposes of the research, farms were appropriately numbered in order to organize the collected information and then used for the analysis.

Each dairy farm was visited once, having made an appointment with the owner in advance. The visit to each farm was carried out according to the same schedule of tasks: collecting data about the farm, identifying technical problems in the barn and taking photos (recording details of the equipment of the barn and cows). A questionnaire was prepared for the implementation of the first two tasks. The informational part of the questionnaire included data on the dairy farm, provided by the farmer and supplemented by reports from the milk recording system. The following information was collected: the number of cows in the herd, the number of other animals in the cattle group (calves, heifers), production data (annual milk yield per cow), the amount of milk delivered to the dairy plant (monthly and annually) and the type and number of animal health problems in last 12 months. Information on animal health problems included mastitis, laminitis and hoof problems, and other health aspects. On most farms, cow hoof problems have been assessed on the basis of hoof-trimmer reports. The hoof-trimmers visited the farms twice a year. Some farmers performed hoof trimming themselves and were aware of hoof problems in their herd. Information on other health problems was taken from documentation kept individually for each of the cows on the farm. This documentation is supplemented monthly by the animal technician when the farms are in the cows’ milk performance control system. Data on some of the health aspects of cattle (ketosis, postpartum problems) also came from the documentation of veterinary visits to farms. Mastitis in dairy herds was identified when the SCC (somatic cell count) exceeded 400,000 cells per ml. Available mastitis tests were used to detect mastitis in farms. In farms with milking parlors, detection of mastitis was supported by properly equipped herd management systems. The identification of dairy cattle diseases was carried out by veterinarians in accordance with veterinary practice.

The informational part of the questionnaire also described some details of the barns on each farm, e.g., number of doors and windows, number of feeding alleys, manure/slurry removal and storage system. In order to calculate the usable area, the basic dimensions of the barn and its individual internal parts were measured. Detailed measurements were made with a laser meter (measuring range 200 m, measuring accuracy 0.1 m) with an optical system of a fixed reference point. To calculate how old the barns were, farm owners were also asked when (in what year) they were built.

The observations of the technical condition of the barns and the identification of damages and construction errors were carried out by two people. During the observation in each barn, these people cooperated with each other, discussing the noticed damages and construction errors. On the basis of joint findings, information on damages and errors was recorded in the questionnaire. One of the people participating in this part of the research (and at the same time co-author of the article) had over 15 years of work experience in a company specialized in equipping facilities for livestock, including dairy cattle.

Based on the data on the number of cows in the farms visited, the average, as well as minimum and maximum number of cows in tie-stall and freestall system barns were compiled (Table 1). These data in descriptive statistics were supplemented with information on the average annual milk yield per cow in herds kept in the two considered housing systems. Data on milk yield per cow in the case of some farms came from the national milk registration system, while for the remaining farms, they were obtained from dairies purchasing milk from farms and supplemented with information on the farm’s own consumption.

Most farms (n = 35) milked cows twice a day, while in three farms equipped with automatic milking systems (AMS), the average number of milkings was 2.8 times a day. Farms other than those equipped with AMS used bucket/pipeline milking systems and milking parlors for freestall barns (Table 2). The visited farms also differed in terms of the lying conditions created for dairy cows. All farms with the freestall housing system kept their cows throughout the year in the barn (most of the barns with an exit to the outer paddock), while the farms with tie-stall housing systems took into account the organizational variant of grazing cows in the period May–October and their stay in the barn for the rest of the year.

For the purposes of the research, in particular data analysis and discussion of the results, four areas (zones) were distinguished in the barns, i.e., the lying area (LA), the social area (SA), the feeding area (FA) and the milking area (MA). The number of damaged technical elements and poorly designed technical parts was identified in individual zones of each of the barns. Apart from the quantitative assessment of technical problems, the qualitative assessment of technical defects was also taken into account by describing each case of failure. Generally, we only took into account visible damage, so, for example, in the milking area (MA), we did not assess operating problems such as incorrect vacuum and malfunction of the pulsator, leaks, and operational wear of the milking cluster. For example, in the lying area, we only assessed the damage and wear of construction elements, but we did not take into account whether the neck-rail is set at the appropriate height and the lying stall has the appropriate width. Gaworski and Boćkowski [52] presented the assessment of the technical parameters of construction elements in individual areas of the barn. This assessment was based on the proposed method of comparing the current housing conditions with those recommended in the production of dairy cattle.

The key stage of the research was the detailed identification of equipment damage and design errors in barns with tie-stall housing system and freestall housing system (Table 3). Equipment damage was interpreted as physical wear of the surface and other parts of the equipment as a result of long-term use and insufficient quality of construction materials. Design and implementation errors, on the other hand, concerned solutions in the barn that were inconsistent with the construction rules, were incorrectly installed and without appropriate security elements. In barns with a freestall housing system, the social area is identified as a place for cattle walking.

Statistical analysis for collected data was performed using the Statistica v.13.3 software [53]. The descriptive statistical indicators, i.e., mean and standard deviation were determined for the assessed cow herds. The comparison of data obtained in the dairy farms with two different, i.e., tie-stall and freestall housing systems was carried out with the use of Mann–Whitney test, including significance level α = 0.05. Detailed data analysis included the calculation of Spearman’s rank-order correlation for the variables included in the study. Developing the descriptive statistical indicators, the mean values, standard deviation (SD), skewness and kurtosis were calculated for two groups of data, i.e., barn with animals and technical problems in four areas.

## 3. Results and Discussion

**Figure 1:**
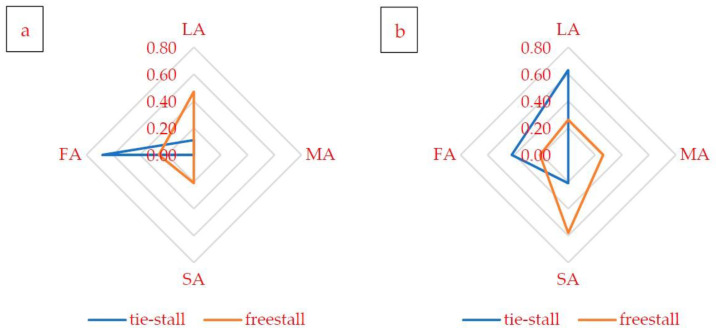
Average (calculated) amount of damage (**a**) and design errors (**b**) per one barn and each zone, including: lying area (LA), milking area (MA), social area (SA) and feeding area (FA), for barns with tie-stall and freestall housing system.

In the first stage of presenting the state of technical equipment in the barns, the number of instances of found damage and design errors was summarized. The calculated average number of instances of damage and design (construction) errors per one barn and each zone (lying, milking, social and feeding area) is shown in Figure 1, separately for barns with tie-stall and freestall housing systems.

The most damage per one barn (0.68) was found in the feeding area (FA) of objects with a tie-stall housing system (Figure 1a). In the same area (FA), but in barns with a freestall housing system, the average number of instances of damage per object was 0.26. In barns with a freestall housing system, the most damage was found in the lying area (LA), i.e., 0.47 instances of damage per barn on average.

When analyzing the data on the number of design errors (Figure 1b), it is worth paying attention to the differences in the distribution of results in the analyzed areas (LA, MA, SA and FA) between barns with tie-stall and freestall housing systems. In two areas (LA and FA) in tie-stall barns, we found a higher number of construction errors compared to barns with alternative housing systems; in the other two areas (MA and SA), the greater number of errors per barn occurred in buildings with the freestall system. The highest number of construction errors (0.63 per barn) was found in the lying area (LA) of buildings with the tie-stall housing system and in the social area (SA) of buildings with the freestall housing system, where the number of construction errors was 0.58 per barn.

**Figure 2:**
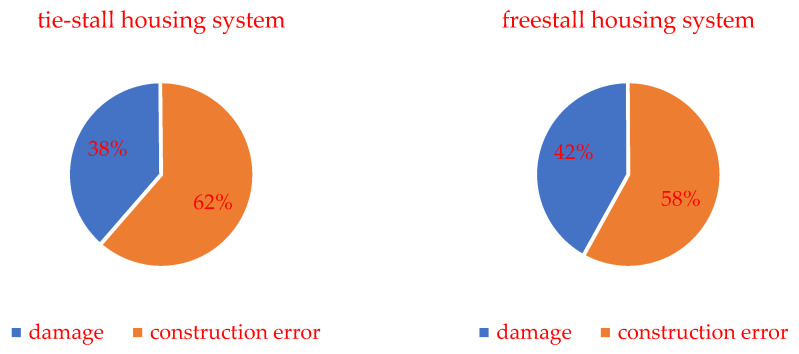
Percentage distribution of damage and construction errors in visited barns with tie-stall and freestall housing system.

Taking into account damage and construction errors as general technical problems, their percentage share was determined on the basis of research results. The distribution of results (percentage share) for the barn group with tie-stall housing system and freestall housing system is shown in Figure 2.

Both in the barn with tie-stall and freestall housing systems, a higher percentage of design errors than damage was found, with the difference being that in barns with a tie-stall housing system, the share of construction errors was higher (62%) compared to barns with a freestall housing system (58%). Accordingly, a higher percentage of damage was found in barns with a freestall housing system compared to barns with a tie-stall housing system.

Data collected in the group of barns with a tie-stall housing system showed that in each farm, technical problems were found in the barns. Including 19 barns, in total, 39 technical problems were identified. The lowest and highest number of technical problems in individual barn with tie-stall housing system amounted to one and four problems, respectively. There were six barns with one technical problem and two barns with four technical problems. The structure of the mentioned total number of technical problems included the following: 14 problems in the lying area (in 11 barns), no problems in the milking area, 4 in the social area (in 4 barns) and 21 problems in the feeding area (in 15 barns). The cow herd size in two barns with a tie-stall housing system and the highest number of technical problems (four problems) included 26 and 33 dairy cows.

Considering data collected in the group of barns with a freestall housing system, it was found that three farms were characterized by a lack of technical problems in the visited barns, while the remaining 16 barns included 42 technical problems in total, i.e., on average, 2.63 problems per barn (in farms with a tie-stall housing system, there were, on average, 2.05 technical problems per barn). For the mentioned group of 16 barns with a freestall housing system, the lowest and highest number of technical problems in individual building amounted to 1 and 5 problems, respectively. There were three barns with one technical problem, and there was one barn with five technical problems. The structure of the mentioned total number of technical problems included the following: 14 problems in the lying area (in 8 barns), 4 in the milking area (in 4 farms), 15 in the social area (in 13 barns) and 9 problems in the feeding area (in 9 barns). The cow herd size in individual barns with a freestall housing system and the highest number of technical problems (five problems) included 50 dairy cows, while three farms characterized by a lack of technical problems were keeping 33, 82 and 110 dairy cows.

Data in the group of technical problems (Table 4) were presented by the average number of cases per one barn. The average barn age was given in years, while the average usable area was given in m^2^ per barn.

The descriptive statistics concerning two groups of barns (Table 4) show some characteristic differences between the objects with a tie-stall and freestall housing system. At the period of investigation, barns with a tie-stall housing system were about 3.5 times older than barns with a freestall housing system. The majority of dairy farms with tethered systems are old barn buildings which are not suitable to keep today’s large-framed dairy cows [54]. Generally, for the past few decades, dairy production in Poland has been based on family farms, and such dairy farms earlier preferred primarily a tie-stall housing system because of the small cow herd size. At the current age among new and modernized barns dominate buildings with freestall housing system as a result of tendency to increase cow herd size and access to EU financial support. Data collected in the presented investigations coincide with such trends in the field of Polish dairy farms development. Lack of space, accessible equipment, sometimes convenience and some economic reasons motivate many farmers to keep dairy cows tethered. The tie-stall housing system is still widely used for dairy cows in many countries. In Europe, between 20% and 80% of dairy cows are tethered, at least during the winter [55].

The other barn data (Table 4) confirmed not only a bigger cow herd size in the farms with a freestall housing system but also a larger usable area per barn with such a housing system. On the other hand, when calculating and comparing the usable area per one cow, it is possible to indicate similar values for barns with a tie-stall and freestall housing system, i.e., 11.69 and 12.89 m^2^ per one animal, respectively. Such a category of area (usable area) shows general space for many activities concerning dairy production in the barns. The different categories of areas in the cowshed are part of the descriptive statistics of research objects. In the studies of von Keyserlingk et al. [56], the area per cow in a pen was taken into account. For the assessed dairy farms in the USA and Canada, this area ranged from 4 to 14 m^2^ per cow.

Older barns with a tie-stall housing system showed a lower average number of identified technical problems (2.05 problems per barn) than objects with a freestall housing system (2.21 technical problems per barn). There was also completely different distribution of technical problems in particular areas, when compared to barns with a tie-stall and freestall housing system. In the first case, i.e., tie-stall system, more than half of technical problems were identified in the feeding area (FA): 1.11 vs. 2.05 (total technical problems per barn)—Table 4. In the barns with a freestall system, the highest average number of technical problems per one barn was found in the lying area (0.74) and social area (0.79).

A lower average number of technical problems in older barns with a tie-stall system when compared with those with freestall system is surprising, so it seems to be important to develop additional analysis of data. There was, on average, one technical problem per 166 m^2^ of usable area in barns with a tie-stall system and one technical problem per 496 m^2^ of usable area in barns with a freestall system. It means that it was one technical problem to at least a threefold-smaller usable area in barns with a tie-stall system, when compared with freestall system barns. Additionally, when we include older-aged barns with a tie-stall system, this can provided an explanation as to why these objects represent a higher intensity of technical problems. The intensity of technical problems can be interpreted, in this case, as the mentioned number of technical problems per usable area or area only for animals in the barn. Put another way, it is possible to extend the analysis of the data for cow herd size and calculate the number of technical problems per one animal kept in barns with a tie-stall and freestall system. There were 0.07 technical problems per cow in barns with a tie-stall housing system and 0.03 technical problems per cow in barns with a freestall housing system. This way, it is possible to consider if differences in housing system organization can help to decide about risk in the contact of animals with technical infrastructure. A higher risk can be identified for barns with a tie-stall system, but on the other hand, the calculated values, i.e., 0.07 and 0.03, are very low, and risk problems should be interpreted circumspectly.

To better recognize the risk troubles, it can be valuable to develop quality aspects of technical problems in the investigated dairy barns. In barns with a tie-stall housing system, the most technical problems were identified in the feeding area (FA). We have found in this area the following problems: concrete losses in the manger (in 10 barns) and wall separating the manger from the lying area (1 farm), lack of individual drinking bowls (water delivered for cows in manger for feed—1 farm), malformed manger (2 farms), damaged drinking bowl cut-off valves (1 farm), misplaced pipe with water on the wall between lying stall and feeding alley (2 farms), improper fixed individual drinking bowls (1 farm) and chains for cows tethering (1 farm). In one farm, an additional metal bar (diameter of 2.5 cm) was identified on the wall separating the manger from the lying area. The most common problem in the feeding area, i.e., concrete losses in the manger, could be the effect of improper quality of material used for concrete. On the other hand, De Belie et al. [57] indicated that the reason for concrete degradation is the specific aggressive conditions occurring on floors in animal houses; chemical components from feed residues can damage the concrete floor surface as well as the surface of the manger.

The second zone with the highest number of technical problems in barns with a tie-stall system was the lying area (LA). The most commonly observed technical defaults and mistakes in the lying area were: improperly installed or damaged chain clamping (5 farms), a horizontal pipe in front part of the stall (3 farms), additional rods in front part of stall (1 farm), lack of partitions (1 farm), vertical posts in lying area (2 farms), and a lack of difference in level of lying surface and rear manure channel (1 farm). A considerable part of the problematic elements in the lying area included metal objects. Generally, metal objects in the barn are exposed for enhancing corrosion as a result of high humidity and temperature, high concentrations of aggressive gases, acids, and salts, but also mechanical destruction [58]. The mentioned mechanical destruction was associated with durability of barn equipment, while our study emphasized importance of technical/mechanical problems in the field of animal comfort and safety in the barn. Of course, corrosion in the livestock building can also show influence on animal comfort, but such an effect will be considered in the other investigations.

For comparison, assessing quality aspects of technical problems in the lying area (LA) in barns with a freestall housing system, the following technical troubles were recognized: neck-rail hogging and lack of clamping elements (4 farms), a lack of partition mounting elements (4 farms), the constructional pillar put in the lying area (1 farm), the gate pillar put in the lying area (1 farm), the lack of neck-rail partly in the stalls (1 farm), badly mounted rubber mats on the lying stalls (1 farm), some cement bricks put in the lying zone (1 farm), incorrect sizes of lying stalls (1 farm) and an additional metal rod at the fence (1 farm). In some barns, we have found that the terminal part of some neck-rails was too long (beyond the extreme lying stall) and not covered by protective caps, so it could be source of animal injuries and other health hazards. The collected data and observations in the lying area showed that except for health hazards, cows in barns with a freestall system can be exposed to decreased comfort as a result of some additional equipment put in lying stalls transferred into a smaller area for lying, incorrectly designed and managed facilities, wear to technical elements and others. So far, the problem of cow comfort in the lying area was considered in many studies in association with some factors creating conditions of rest and managed as well as designed by man. Fregonesi et al. [14] investigated the effect of bedding quality on cow preferences and found that dairy cows show a clear preference for a dry lying surface, and they spend much more time standing outside the stall when only wet bedding is available. Tucker et al. [59], by testing three types of freestall surfaces, indicated that a freestall surface can affect both stall preferences and stall usage, and mattresses are less preferred. Drissler et al. [13] documented that lying times reduced with decreasing bedding, such that cows using the stalls with the least amount of bedding spent less time per day lying down than when housed with access to freestalls filled fully with sand. Tucker et al. [60] indicated that cow comfort expressed by lying time can be created by freestall dimensions. These dimensions decide about area (in m^2^) and space (in m^3^) accessible in lying area for cows.

The highest skewness coefficients, i.e., asymmetry measures of the analyzed research data, were found in the lying area (LA) and social area (SA) of barns with a tie-stall housing system, as well as the milking area (MA) of barns with a freestall housing system (Table 4). In a barn with a tie-stall system, the highest kurtosis (2.17) was found in the category of problems in the lying area (LA)—Table 4; a positive value of kurtosis indicates that the intensity of the extreme values is greater than for the normal distribution. The largest negative kurtosis value for the barn with the freestall system (−2.24) was identified for problems in the feeding area (FA); a negative value of kurtosis indicates that the intensity of the extreme values is smaller compared to the normal distribution.

The results of our study show that accessible space in the lying area can be disrupted by improperly designed and equipped stalls, so it seems to be significant to inspect barns in a more correct way at the stage of putting these buildings into operation.

Both the wear of technical barn equipment and any errors at the construction and building stages can be a source of threats and health problems for livestock animals. Information on animal health problems was collected during visits to the farms studied. A summary of this information is provided in Table 5.

Of course, not all cow health problems result directly from the technical imperfections of buildings. Mastitis had the largest share in the overall structure of health problems found in the visited barns (Table 5). In many studies, mastitis in a herd of dairy cows is considered in association with the use of key technical equipment for dairy farms, i.e., milking systems. This applies to both milking systems used in barns with a tie-stall system and barns with a freestall system equipped with milking parlors [61] and milking robots [62]. As indicated by the authors of the cited publications, in the case of mastitis and other diseases, many risk factors characteristic of farms with conventional milking systems are also noted on farms equipped with milking robots. Milking devices in connection with cow hygiene are key factors determining the risk of mastitis, but in larger farms with an AMS (automatic milking system), the udder health problem may require more attention compared to smaller farms (with a smaller herd size) with AMS [62]. Mastitis can be a consequence of a malfunctioning milking installation, as well as improperly adjusted working parts of milking parlors, which is why it is recommended to take specific practical measures to protect against the risk of disease [63].

The health problems of the dairy herd are among the important arguments in the discussion on the evaluation of dairy production in barns with a tie-stall and freestall housing system. However, these are not the only arguments. In numerous studies comparing tie-stall and freestall systems, particular emphasis is placed on animal welfare. In a study involving 36 dairy herds, Seo et al. [64] calculated the Animal Needs Index (ANI) and, on this basis, found that the herds kept in the freestall system had a significantly higher (and therefore more favorable) ANI score compared to the herds in the tie-stall system barns. The authors also pointed out the importance of a proper approach to subjective evaluation criteria, including cleanliness and slipperiness of floors, when comparing two dairy housing systems. Beaver et al. [65] compared tie-stall systems with less restrictive types of dairy housing systems based on a systematic review of the scientific literature and considered three welfare criteria, i.e., health and biological functioning, natural behavior, and affective state. In these considerations, the main emphasis is placed on the comparisons of cattle housing systems in terms of biological production potential. This potential is created by the animals, their production data, health problems and other indicators related to the evaluation of dairy herds kept in tie-stall and freestall housing systems. Gaworski et al. [26] highlighted a significantly lower number of cases of udder diseases, hoof diseases in herds and problems with insemination of cows kept in the freestall housing system. Keeping cows in the tie-stall system is subject to critical assessment due to the risks associated with lowering animal welfare, which may translate into a gradual reduction in this cattle housing system [66] in favor of the freestall system. On the other hand, it is possible to cite the opinion [67] that a well-designed tie-stall can contribute to the reduction in physical and behavioral problems in the absence of full freedom of animals, characteristic of the freestall system.

Table 6 presents the results of the Spearman’s rank order correlation analysis, taking into account the main factors (variables) in the research.

The highest coefficient of Spearman’s rank correlation (0.849) was found when comparing the herd size with the usable area (Table 6). In this case, the high correlation coefficient confirms the key role attached to meeting the requirements related to providing the animals with sufficient living space in the barn. The recommendations in this regard [68] clearly define the area per one animal in the barn. In the case of recommendations and standards, this area is mainly considered in relation to the lying and feeding area. In our study, we took into account the usable area, which includes additional areas in the barn, important for animals and the efficiency of dairy production. The relatively high correlation coefficient (0.645) can also be indicated when laminitis is associated with the size of the herd (Table 6). Such a relationship has been highlighted in many studies, including models of clinical laminitis assessments taking into account the size of the herd and other factors (contamination of lying stalls, rubber on the floor) [69]. The age of the barns included in the study was negatively correlated with their usable area and the size of the cow herd. These research results confirmed the general tendency of new barns to become larger facilities with a large number of cows. High concentration of dairy production on farms is a premise for the implementation of modern technologies and technical progress [70]. The number of mastitis cases in a herd of dairy cows was not correlated with the age of the barn. It might seem that in older barns it is more difficult to maintain an adequate level of hygiene, which translates into a higher risk of developing mastitis. DeVries et al. [71] linked barn hygiene with cow hygiene and the risk of increased somatic cells (SCC). They indicated that cow hygiene, especially the udder hygiene, is significantly related to the cleanliness of the cow’s environment.

The results presented in Table 6 show that the most significant correlations were found for the usable area of the barn (six correlations), herd size and laminitis (five correlations), as well as barn age and other hoof problems (four correlations). Associations of various hoof problems with risk factors at herd level were also investigated by Cramer et al. [72]. The authors pointed to the importance of such risk factors in the assessment of hoof problems, such as the bedding depth in the lying stalls (in freestall barns), as well as access to the outside areas, the size of the cow herd and the use of sawdust in the lying stalls (in the tie-stall barns).

Considering the problems in individual areas of the barn, the highest number of significant correlations was found in the social area (SA), with the lowest in the lying area (LA) and milking area (MA) (Table 6). The significant correlation of the problems in the lying area (LA) with the other hoof problems can be considered justified. Poorly designed and improperly maintained lying stalls can cause hoof problems [73].

There are some limitations in carrying out studies involving the assessment of barn infrastructure damage and design errors taking into account association with selected dairy health aspects. Data on animal health problems came from a variety of sources including reports from zootechnicians, veterinarians and farmers. The scope of animal health diagnostics, the accuracy of compilation of health data and their detailed description remains an issue. The quality of the collected health data translates into the results of the analysis and the resulting conclusions. Reliable assessment of damage to barn equipment, and especially design errors, requires specific knowledge and experience of people conducting research in livestock facilities. In this case, the aforementioned experience may be professional experience, including the design of livestock buildings or the revitalization of barns based on the assessment of their technical condition and equipment.

The results of the comparison of two groups of facilities, i.e., barns with tie-stall and freestall maintenance systems based on the Mann–Whitney U test are presented in Table 7. Statistically significant differences (marked in red) for the considered variable (housing system) were found in the case of seven compared values.

The Mann–Whitney U test, used to verify the hypothesis of the insignificance of differences between the medians of the studied variable in two populations, provided information to compare the two housing systems. Among the features directly affecting cows, significant differences between tie-stall and freestall barns were found for laminitis, hoof problems and other health problems. The differences between tie-stall and freestall barns in terms of cow health were also noted by Witkowska and Ponieważ [74]; the variable of assessment was the prevalence of diseases, including mastitis. The prevalence of diseases in connection with the cow housing system was also noted by Simensen et al. [75], indicating that the results of the observations depend on the number of cows in the herd. Comparing the herds of cows in barns with different housing systems, Gaworski et al. [26] indicated their significant differentiation in the case of udder diseases, hoof diseases and metabolic problems. In these studies, the authors also noted the significant differentiation of cow herds in terms of fertility problems. Comparing the two cow housing systems, Praks et al. [76] pointed out that in freestall barns there is a higher risk of foot diseases and a lower prevalence of mastitis compared to tie-stall systems. Animal health problems are considered in some studies in relation to welfare [77]. The cow welfare assessment is therefore an important link in the comparison of different housing systems in barns, including tie-stall and freestall systems. In the studies of Popescu et al. [78], based on the use of the Welfare Quality protocol, significant differences were identified between the two housing systems (tie-stall and freestall) for most parameters within the four welfare principles. The study concluded that the loose system is better for cattle in terms of feeding, housing and behavior.

The most important challenges of modern dairy production include ensuring appropriate living conditions and animal welfare, which are part of the effective, sustainable development of the farming system [79]. This sustainable development includes livestock facilities and their technical equipment, which imply technical and technological progress in dairy production [80,81]. Research covering the identification of problems with technical equipment in barns is part of the process of improving knowledge about dairy production. This knowledge may lead to the formulation of a vision of an ideal dairy farm. In this vision, cows and other groups of animals should have access to high-performance technical equipment ensuring comfort and safety. Farms with barns providing comfort and safety for cattle and working people are part of the trends of global changes in the economies of many countries [82].

## 4. Conclusions

The intention of the research was to indicate that apart from the previously known criteria, the two basic cattle housing systems, i.e., tie-stall and freestall, can also be compared in terms of technical damage to livestock buildings and construction errors. The question is how damage to livestock buildings and construction errors can affect the comfort, welfare and safety of dairy cattle, especially in contact with the technical equipment of barns.

The approach involving the assessment of damage to the technical equipment of barns and design errors can be practically used as one of the factors in the evaluation of farms applying for funding for the improvement of livestock buildings and livestock production conditions. A practical tool for assessing the production conditions in an animal facility is a proposal for use by the agricultural advisory service and units responsible for the assessment of animal welfare conditions. The presented approach to research may become an inspiration for the scientific development of a method of monitoring the technical condition of farm equipment in connection with the improvement of animal welfare.

The gradual degradation of livestock buildings and their technical equipment is obvious. The conducted research has shown that the rate of this degradation may differ not only between buildings with two cattle housing systems, but also between zones (lying, feeding, milking and social), which are important for the vital functions of animals. It seems justified to develop further research in which the degradation of barns and their technical equipment as well as design errors can be linked to the assessment of the welfare of dairy cattle.

## Figures and Tables

**Table 1:** Production data of cow herds in the investigated dairy farms.

Description	Dairy Farms with Housing System Type:	
	Tie-Stall	Freestall
Number of visited farms	19	19
Average cow herd size ± SD	29 ± 14	85 ± 38
Min./max. cow herd size	8/60	30/150
Average annual milk yield per cow ± SD [kg cow^22121^ year^−1^]	5261 ± 1329	6749 ± 1483
Min./max. cow milk yield [kg cow^−1^ year^−1^]	3333/8400	3900/9000

Explanation: SD—standard deviation.

**Table 2:** Description of milking and lying areas in the investigated dairy farms.

Description	Dairy Farms with Housing System Type:	
	Tie-Stall	Freestall
Number of visited farms	19	19
Milking system (m.s.)/ milking parlor (m.p.) used on the farm	bucket m.s. × 6 farms pipeline m.s. × 13 farms	herringbone m.p. × 11 farms side by side m.p. × 5 farms AMS × 3 farms
Lying area in the barn	rubber mat × 1 farm shallow straw bedding × 18 farms	rubber mat × 9 farms shallow straw bedding × 10 farms

Explanation: AMS—automatic milking system; m.s.—milking system; m.p.—milking parlor.

**Table 3:** Technical condition of the equipment and design errors assessed in four zones of barns with a tie-stall housing system and freestall housing system.

	The Zone in the Barn	Technical Equipment Assessed in the Zone	Identified Damage to Barn Equipment	Identified Design and Implementation Errors
Tie-stall housing system	Lying area (LA)	concrete floor, rubber mats, partitions, manure channel, tether	bent metal parts and pipes, damaged chain fastening, defects in concrete in the floor and slurry channel, damaged mats, broken mats	ribbed bar at the front of the stall and its incorrect attachment, no partitions, additional pipe at the front of the stall, incorrect chain length and fastening, no lowering of the manure channel in relation to the stall level, partition with an additional vertical pipe
	Milking area (MA)	milking pipes and connectors	bent pipes, damaged vacuum gauge, damaged milking cluster fittings	incorrect installation of vacuum and milk lines
	Social area (SA)	concrete floor in a walking alley, concrete grid for collecting faeces	concrete damage in the floor and grid	additional concrete threshold in the corridor, pipe installed across the corridor, badly made partitions at the end of the row with stalls
	Feeding area (FA)	feeding alley, manger, separating wall, feed ladder, drinking bowl	concrete defects in the manger and fracture, concrete defects in the feeding alley, folded metal parts and pipes, feed ladder defects, drinking bowl defect, damaged drinkers valves	no drinking bowls (water intake from a concrete manger), no running water installation, no level difference between the manger and lying stalls, water pipe on the wall separating the feed alley from the lying stalls, incorrect mounting of the drinkers, metal rod in the feed wall
Freestall housing system	Lying area (LA)	concrete floor, rubber mats, partitions, neck-rails, brisket-boards, curbs	bends of metal elements, no fasteners and securing elements on neck-rails, no fastening elements for partitions, no neck-rails in some lying stalls	additional bars at the pen’s fence, construction posts in the lying area, incorrectly installed rubber mats, the post of the pass gate in the lying area
	Milking area (MA)	concrete floor in the milking parlor, concrete floor in the waiting area, entrance and exit gates, fencing structure, elements of milking equipment	damaged floor in the milking parlor, broken entrance gate to the milking parlor	too high threshold at the entrance to the milking parlor, improper separation (with a metal pipe) of the entrance to the milking parlor, faulty entrance gate, incorrect width of the entrance to the milking parlor
	Social area (SA)	concrete floor in the walking alley, concrete grid for faeces collecting, brush	damaged floor in walking alleys, damaged metal fillings of entry gates, wear and improper operation of the cow brushes	no end caps in the horizontal mounting pipe protruding from the row of stalls into the walking alley, too high threshold at the exit to the external paddock, water system valves not properly secured, improperly mounted brushes
	Feeding area (FA)	feeding alley, manger, separating wall, feed ladder, drinking bowl	damaged drinking bowl valve, concrete losses in the feeding alley, losses in the feed ladder	unprotected metal bars and wooden elements at the drinking bowls, sharp drinking bowls’ edges, badly mounted salt licks, protruding metal rods on the feed wall

**Table 4:** Descriptive statistics concerning technical problems in the investigated dairy farms, including number of technical problems per barn.

	Data	Barns with Tie-Stall Housing System				Barns with Freestall Housing System			
		Mean	SD	Skewness	Kurtosis	Mean	SD	Skewness	Kurtosis
Barn	Barn age (years)	21.68	12.40	0.12	−1.48	6.16	4.22	0.64	−0.04
	Usable area (m^2^)	340.26	138.35	0.17	−1.03	1095.21	289.09	−0.27	−0.89
	Herd size (cows)	29.11	14.00	0.68	0.22	85.00	37.66	0.16	−0.91
	Usable area per cow (m^2^ cow^−1^)	11.69	6.40	1.69	1.77	12.89	6.13	1.39	1.78
Technical problems	Problems in LA	0.74	0.81	1.25	2.17	0.74	0.93	0.59	−1.68
	Problems in MA	0.00	0.00	−	−	0.21	0.42	1.54	0.42
	Problems in SA	0.21	0.42	1.54	0.42	0.79	0.63	0.17	−0.31
	Problems in FA	1.11	0.74	−0.17	−1.00	0.47	0.51	0.11	−2.24
	Technical problems together	2.05	0.97	0.70	−0.20	2.21	1.47	0.06	−0.77

Explanation: LA—lying area; MA—milking area; SA—social area; FA—feeding area; SD—standard deviation.

**Table 5:** Descriptive statistics on health problems in the investigated dairy farms, including the number of health problems per barn during the last 12 months.

	Health Problems	Barns with Tie-Stall Housing System				Barns with Freestall Housing System			
		Mean	SD	Skewness	Kurtosis	Mean	SD	Skewness	Kurtosis
Health problems	Laminitis	1.05	3.42	4.18	17.82	6.26	5.16	0.93	1.13
	Other hoof	0.74	2.31	4.01	16.67	5.89	7.16	0.91	−0.42
	Mastitis	14.74	7.00	0.49	−1.19	11.42	11.28	0.88	−0.38
	Other health problems	1.16	3.13	2.75	6.31	0.00	0.00	−	−
	Total health problems	17.68	9.62	0.58	−1.09	23.58	10.55	0.27	−0.15
	Health problems per cow	0.68	0.34	0.54	0.29	0.34	0.27	0.50	1.04

Explanation: SD—standard deviation.

**Table 6:** Spearman’s rank order correlations for the main factors (variables) in the research; marked results (in red) are significant with *p* < 0.050.

	Barn Age	Usable Area	Herd Size	Problems in:				Laminitis	Other Hoof Probl.	Mastitis	Other Health Probl.
				LA	MA	SA	FA				
Barn Age	1.000	−0.627	−0.388	0.230	−0.317	−0.295	0.502	−0.383	−0.177	0.089	0.126
Usable Area	−0.627	1.000	0.849	−0.013	0.289	0.390	−0.338	0.638	0.400	−0.182	−0.123
Herd size	−0.388	0.849	1.000	0.099	0.153	0.333	−0.223	0.645	0.543	−0.269	−0.130
Probl. LA	0.230	−0.013	0.099	1.000	−0.119	−0.015	0.029	0.124	0.364	−0.093	−0.068
Probl. MA	−0.317	0.289	0.153	−0.119	1.000	0.339	0.000	0.269	0.032	−0.071	−0.117
Probl. SA	−0.295	0.390	0.333	−0.015	0.339	1.000	−0.212	0.231	−0.040	0.137	−0.302
Probl. FA	0.502	−0.338	−0.223	0.029	0.000	−0.212	1.000	−0.225	−0.102	0.238	0.351
Laminitis	−0.383	0.638	0.645	0.124	0.269	0.231	−0.225	1.000	0.591	−0.351	−0.165
Other hoof probl.	−0.177	0.400	0.543	0.364	0.032	−0.040	−0.102	0.591	1.000	−0.265	−0.125
Mastitis	0.089	−0.182	−0.269	−0.093	−0.071	0.137	0.238	−0.351	−0.265	1.000	0.272
Other health probl.	0.126	−0.123	−0.130	−0.068	−0.117	−0.302	0.351	−0.165	−0.125	0.272	1.000

Explanation: LA—lying area; MA—milking area; SA—social area; FA—feeding area; Probl., probl.—problem.

**Table 7:** Results of the Mann–Whitney U test for the variable: Housing system; marked results (in red) are significant with *p* < 0.050.

	Median ts	Median fs	Z	*p*
Barn age	16	5	4.09	0.000
Usable area	325	1050	−5.23	0.000
Herd size	26	85	−4.53	0.000
Probl-LA	1	0	0.18	0.861
Probl-MA	0	0	−1.09	0.274
Probl-SA	0	1	−2.60	0.009
Probl-FA	1	0	2.44	0.015
Total-tech	2	2	−0.38	0.704
Laminitis	0	5	−3.91	0.000
Other-hoof	0	2	−2.07	0.038
Mastitis	12	8	1.65	0.099
Other-probl.	0	0	1.09	0.274
Total-probl.	13	21	−1.65	0.099

Explanation: ts—tie-stall housing system; fs—freestall housing system; Z—the value of the Mann–Whitney U test; *p*—significance level calculated for the test value; probl.—problems; Total-tech—total technical problems.

## Data Availability

Not applicable.
